# Protein ligand-specific binding residue predictions by an ensemble classifier

**DOI:** 10.1186/s12859-016-1348-3

**Published:** 2016-11-17

**Authors:** Xiuzhen Hu, Kai Wang, Qiwen Dong

**Affiliations:** 1 College of Sciences, Inner Mongolia University of Technology, Hohhot, 010051 People’s Republic of China; 2 College of Animal Science and Technology, Jilin Agricultural University, Changchun, 130118 People’s Republic of China; 3 Institute for Data Science and Engineering, East China Normal University, Shanghai, 200062 People’s Republic of China; 4 Key Laboratory of Network Oriented Intelligent Computation, Harbin Institute of Technology Shenzhen Graduate School, Shenzhen, Guangdong 518055 People’s Republic of China; 5 Present Address: School of Computer Science and Software Engineering, East China Normal University, #3663, North Zhongshan RD, Shanghai, 200062 China

**Keywords:** Binding residue prediction, Ensemble classifier, Protein function

## Abstract

**Background:**

Prediction of ligand binding sites is important to elucidate protein functions and is helpful for drug design. Although much progress has been made, many challenges still need to be addressed. Prediction methods need to be carefully developed to account for chemical and structural differences between ligands.

**Results:**

In this study, we present ligand-specific methods to predict the binding sites of protein-ligand interactions. First, a sequence-based method is proposed that only extracts features from protein sequence information, including evolutionary conservation scores and predicted structure properties. An improved AdaBoost algorithm is applied to address the serious imbalance problem between the binding and non-binding residues. Then, a combined method is proposed that combines the current template-free method and four other well-established template-based methods. The above two methods predict the ligand binding sites along the sequences using a ligand-specific strategy that contains metal ions, acid radical ions, nucleotides and ferroheme. Testing on a well-established dataset showed that the proposed sequence-based method outperformed the profile-based method by 4–19% in terms of the Matthews correlation coefficient on different ligands. The combined method outperformed each of the individual methods, with an improvement in the average Matthews correlation coefficients of 5.55% over all ligands. The results also show that the ligand-specific methods significantly outperform the general-purpose methods, which confirms the necessity of developing elaborate ligand-specific methods for ligand binding site prediction.

**Conclusions:**

Two efficient ligand-specific binding site predictors are presented. The standalone package is freely available for academic usage at http://dase.ecnu.edu.cn/qwdong/TargetCom/TargetCom_standalone.tar.gz or request upon the corresponding author.

**Electronic supplementary material:**

The online version of this article (doi:10.1186/s12859-016-1348-3) contains supplementary material, which is available to authorized users.

## Background

The purpose of protein research is to identify and annotate protein functions. Many proteins perform their functions by interacting with other ligands, although only a small portion of the residues are in contact with the ligands. The recognition of binding residues is important for the elucidation of protein functions and drug design applications [1]. Experimental methods to detect the binding residues are often expensive and time-consuming. With the large and increasing number of sequences deposited in various databases, it is valuable to predict the ligand binding sites using computational methods.

During the last decade, much effort has been made towards accurately predicting ligand binding sites [2, 3]. Roughly speaking, these methods can be grouped into the following categories based on the source of the information used [4]: sequence-based methods, structure-based methods and hybrid methods that combine sequence with structure information [5]. The sequence-based methods [6] extract diverse features from the protein sequence directly or indirectly and input the features into a classifier to predict the possibility of binding residues. The most widely used feature is the position-specific scoring matrix (PSSM) generated by PSIBLAST [7]. Other predicted features have also been used, including the predicted secondary structure, predicted solvent accessibility and predicted dihedral angles. Fang et al. [8] demonstrates that PSSM contains most of the information needed for ligand function site prediction. Evolutionary conservation is an important indicator for function-related residues. The Rate4Site method [9] calculates the conservation score based on polygenetic trees and uses the score to detect functionally important regions in proteins with known three-dimensional structures. Capra et al. [10] presented a simple but efficient method that used Jensen-Shannon divergence to estimate sequence conservation. The structure-based methods basically dominate this field [11]. These methods generally use known templates with similar topology structures to find the “pocket” or “cavity” on the structure surface. The template-based methods search homologous structures with global topology; then, the putative binding sites can be transformed after superposition [12, 13]. The homology-derived model is still useful even if the structure of the target protein is not available [14]. The global comparison methods can find templates with similar topology, but the alignment in the binding pocket may not be accurate. The local comparison is sensitive to the binding pocket but has a high false positive rate [15]. The combination of global and local comparisons can obtain robust results, as shown by COFACTOR [16]. The other type of structure-based method searches the surface of the structure to find either a geometry-complementary [17] or energy-favourable [18, 19] region as the possible binding site. The hybrid methods use both sequence and structure information to obtain better predictions. For example, ConCavity [20] integrates the residue conservation scores and the output of other structure-based methods to identify protein surface cavities, and FREPS [21] predicts functional regions by detecting spatial clusters of conserved residues on the protein structure.

Although much progress has been made in computational binding site predictions, many issues with the current methods require further investigation.

First, many approaches use three-dimensional protein structures to identify the binding sites. In reality, only a very small proportion of proteins have experimentally solved structures deposited in Protein Data Bank (PDB) [22]. Obtaining structures for many proteins is difficult due to purification and crystallization issues. In contrast, available sequences [23] are exponentially increasing due to the advance of high-throughput sequencing techniques. Although structure models can be obtained using template-based [24] or *ab initio* structure prediction [25], the quality of the model has an important influence on the confidence of the binding site prediction, especially for hard target proteins [26] that do not have homologous templates in the current PDB library. Thus, it is necessary to develop powerful methods for binding site prediction from protein sequence information alone. This study will demonstrate that the sequence-based method is an effective complement when template-based methods fail to obtain a good predicted structure model.

Second, most methods try to obtain all binding sites without carefully checking the differences between different ligands. However, ligands are chemically and structurally different. The assessment of binding site residue predictions in CASP9 [27] suggests that the assessment should be made according to the chemo-type categories of the ligand. The ProBiS-ligands server [28] predicts the types of ligands that can be bound to a given structure. Recently, researchers have paid attention to the differences in ligands, and many ligand-specific methods have been developed to obtain more accurate predictions. For example, Bharat et al. developed VitaPred [29] to predict vitamin-interacting residues, Moreover, nucleotide-binding residues were predicted using SITEpred [30] and ATP binding residue predictions were extensively investigated using many methods [31, 32]. Other ligands have also been explored, such as HEME [33], FAD [34], calcium [35], GTP [36], NAD [37], and zinc [38].

Third, the principle of protein-ligand binding is complicated, and each method can only explore specific binding site information. Thus, the combination of multi-methods can result in better predictions. For example, MetaPocket 2.0 [39] combines eight methods to generate a consensus output for function site predictions. COACH [40] also achieves better predictions by integrating five methods.

In view of the above-mentioned statement, this study will present a robust ligand-specific binding residue predictor. Nine ligands were initially investigated to validate the proposed method. However, the proposed framework can easily integrate other ligand-specific predictors without much revision. First, a sequence-based method called TargetSeq was developed; this method only uses features from the protein sequence. The extracted features include the position-specific scoring matrix, the residue conservation scores, and the predicted secondary structure. These features are inputted into an ensemble classifier that is based on a modified AdaBoost algorithm to tackle the serious imbalance problem between the positive samples (binding residues) and negative samples (non-binding residues). Second, a combined method called TargetCom was developed that integrated the outputs of four well-established methods (COACH [40], COFACTOR [16], TM-SITE [40] and S-SITE [40]). Extensive experimental results show that the combined method outperforms each of the individual methods.

## Methods

### Benchmark dataset and ligands

Most ligand binding site prediction methods use three-dimensional structures from the PDB database [22]. A non-redundant subset for specific or general ligands is obtained as a benchmark dataset after filtering the whole database. However, not all the ligands in PDB are natively bound to the structures. Many ligands are included as additives to help solve the structures. Thus, much effort has been made to filter out the biologically relevant ligands from the PDB structures, and many well-established databases have been developed, such as FireDB [41], LigASite [42], PDBbind [43] and BioLip [44]. Because BioLip is a newly developed and semi-manually curated database, this study uses BioLip as the data source. First, PDB chains with specific ligands are extracted from the BioLip database. If one chain has multiple sites with the same type of ligand, all sites are considered effective. Then, these structures are filtered by keeping only structures with a resolution less than 3.0 Å and a sequence length larger than 50 residues. Redundant structures are removed using the CD-HIT program [45] with a sequence identity threshold of 0.4. Although CD-HIT is extremely fast and is widely used, similarities are estimated by common word counting instead of a sequence alignment. Thus, there are some odd data in which a pair of sequences may be a little higher than the specific threshold. To obtain strict non-redundant benchmark data, the dataset is filtered using the global dynamic programming algorithm of the Needleman-Wunsch alignment.

Nine types of ligands are used here to evaluate the proposed ligand-specific method; these nine ligands are comprised of six small ligands and three large ligands. The small ligands contain four metal ions (BioLip ID: CU, FE, FE2 and ZN) and two acid radical ions (BioLip ID: SO4 and PO4). The large ligands contain two nucleotides (BioLip ID: ATP and FMN) and one HEME. The ligand HEME corresponds to the HEM and HEC ligands in the BioLip database because they are two subtypes of the HEME molecule. The detailed composition of the dataset is given in Table 1.

**Table 1:** Composition of the dataset for the 9 types of ligands

Ligand Categories	Ligand ID^a^	No. Proteins	No. Positive^b^	No. Negative^c^
Metal ions	CU	110	535	38488
	FE	227	1115	73813
	FE2	103	439	34113
	ZN	933	4317	367292
Acid radical ions	SO4	303	2125	99729
	PO4	339	2168	112279
Nucleotides	ATP	261	3631	100848
	FMN	95	1552	30244
HEME	HEM and HEC	228	5821	69155

^a^The ligand ID in the BioLip database

^b^The number of binding residues

^c^The number of non-binding residues

For each ligand, five-fold cross-validation is used to evaluate the performance of the proposed method. The dataset is randomly divided into five parts. One part is used to obtain the test results, and the other four parts are used to train the model. The above process is repeated five times so that each part is tested. The average performance over the five parts is reported as the final cross-validation result.

### Sequence-based method pipeline

**Fig. 1:**
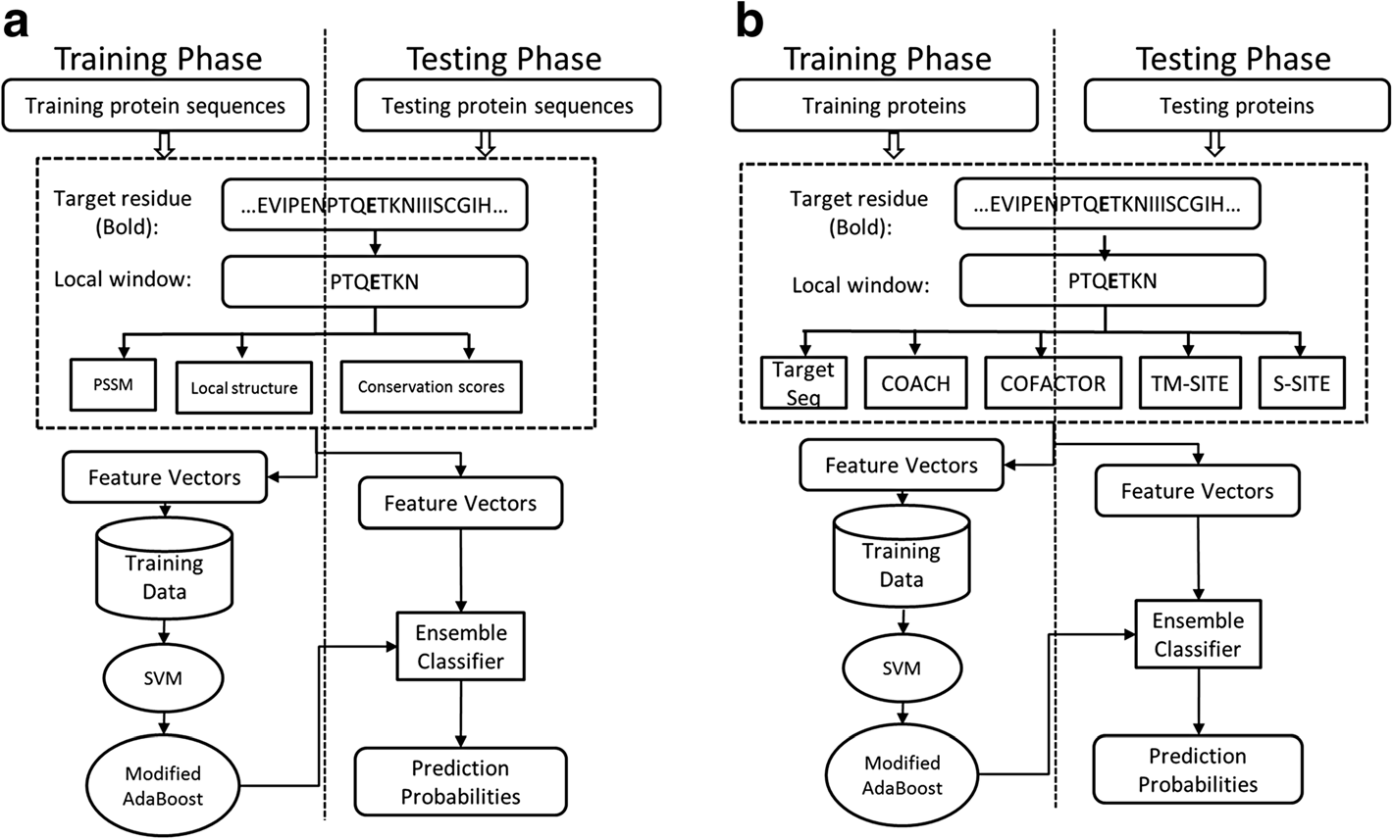
The flowchart of the proposed TargetSeq (**a**) and TargetCom (**b**) methods for protein-ligand binding site prediction

First, we present a sequence-based method named TargetSeq, which only uses information from protein sequences or their variants through a multiple sequence alignment (Fig. 1a). For a target residue in a protein sequence, a sliding window with length *L* is used to extract the protein sequence features including the position-specific scoring matrix, the predicted structure properties and the conservation scores. The target residues are then represented as feature vectors. These vectors are then inputted to support vector machine to get the classifier. Note that to handle the class-imbalance problem, the modified AdaBoost algorithm is used to get the ensemble classifier. For a testing target residue, the same procedure is used to get the feature vector and the ensemble classifier is used to get the probability of binding site. The binding sites are predicted in ligand-specific manner. For each type of ligands, the corresponding ensemble classifier is constructed. The overall flowchart is illustrated in Fig. 1a. Detailed feature encoding and training algorithm are explained below.

### Position-specific scoring matrix

The position-specific scoring matrix (PSSM) contains protein evolutionary information. PSSM has been widely used for many prediction problems in bioinformatics. In this study, the position-specific scoring matrix is generated by running PSI-BLAST [7] on the non-redundant protein dataset (nr) from NCBI with an e-value threshold of 0.001 and iteration time of three. The original PSSM scores are transformed by the following logistic function before they are extracted as features:

1 $$ y=\frac{1}{1+{2}^{-x}} $$

where *x* is the original PSSM value, and *y* is the normalized value. A sliding window with length *L* centred at the target residue is used to extract the PSSM value. The window length is a parameter of the method and needs to be optimized during cross-validation. Due to the distinction of different ligands, each ligand has its own optimal window length as shown in the Results section. Therefore, the number of dimensions of the PSSM features is L*20.

### Predicted structure properties

Previous studies showed that the predicted structure properties were helpful for function site identification. Here, we use the predicted secondary structure, relative solvent accessibility and torsion angles as additional features. The predicted secondary structures are obtained using PSIPRED [46], and a three-dimensional vector with a Boolean value is used to indicate the type of secondary structures defined as alpha-helix, beta-strand, and coil. The relative solvent accessibilities are predicted by ANGLOR [47] which uses the neural network as the classifier, and only one Boolean value is used to illustrate whether the residue is buried (<25%) or exposed (>25%). The backbone torsion angles are also predicted by ANGLOR [47], and the two-dimensional real value is used to show the *φ* and *ψ* dihedral angles. Taking the local window with length *L* into consideration, the number of dimensions of the predicted structure properties is L*6.

### Conservation scores

Residue conservation is a crucial indicator for functionally important residues that has been extensively investigated and well used for ligand binding site prediction. First, the position-specific conservation is calculated by the software implemented by Capra and Singh [10], with two information-theoretic scores [the relative entropy score (RE) and Jensen-Shannon divergence score (JSD)] used as the features. The JSD score has been reported to perform similarly to the Rate4Site algorithm [48] for the identification of functionally important residues, but the JSD algorithm is several orders of magnitude faster than the Rate4Site algorithm. The number of dimensions of the position-specific conservation is L*2. In addition to the above position-specific conservation, we also consider the conservation of the sequence segment within the entire local window. A position weight matrix, which is similar to the PSSM, is constructed based on all sequence segments. The occurrence frequency of each residue in the specific position within the local window is calculated as follows:

2 $$ {p}_{i,j}=\frac{n_{i,j}+\sqrt{N_i}/21}{N_i+\sqrt{N_i}} $$

where *i* denotes the position index within the window, *j* denotes one of the twenty residues plus an additional residue used to denote the unknown residue or the residue outside of the sequence, *n*_*ij*_ is the occurrence number of residue *j* at position *i*, *N*_*i*_ is the occurrence number of all residues in position *i*, and *p*_*ij*_ is the frequency of residue *j* at position *i* and is further normalized by the background frequency:

3 $$ {m}_{i,j}= \log \left(\frac{p_{i,j}}{p_j}\right) $$

where *P*_*j*_ is the background frequency of residue *j* and *m*_*ij*_ is the matrix element of the position weight matrix. A conservation score for a specific sequence segment can be calculated based on the position weight matrix and the sequence of the segment as follows:

4 $$ S=\frac{{\displaystyle \sum_{i=1}^L\left({m}_{i,{s}_i}-{m}_{i, \min}\right)}}{{\displaystyle \sum_{i=1}^L\left({m}_{i, \max }-{m}_{i, \min}\right)}} $$

where *m*_*i,min*_ and *m*_*i,max*_ are the minimum and maximum values, respectively, for position *i* in the matrix, and *s*_*i*_ is the residue type at position *i* for the target sequence segment. The above score can be calculated for the positive and negative samples so that a two-dimensional vector can be obtained as the feature for each sequence segment.

In this study, support vector machine (SVM) is used as the base classifier. SVM is a class of supervised machine learning algorithms that was first presented by Vapnik [49]. SVM has shown excellent performance in practice and has a strong theoretical foundation of statistical learning. Here, the LibSVM package [50] is used as an implementation of the SVM, and the radial basis function is selected as the kernel. The parameter *λ* in the kernel function and the regularization parameter *C* are selected based on the cross-validation.

There are serious class-imbalance problems in ligand binding site predictions (i.e., the number of binding site residues is far lower than the number of non-binding site residues). The traditional machine learning algorithms cannot perform well on these datasets because they are developed on the assumption that the class is balanced. Recently, the ensemble classifier has arisen as one possible way to solve the imbalance problem. The basic idea of the ensemble classifier is to train multiple base classifiers and combine them to obtain a single class label. The AdaBoost algorithm [51] is one of the most representative methods. AdaBoost trains a series of base classifiers by randomly selecting samples from the training dataset. For each round, the misclassified samples are assigned large weights so that they may be re-trained in the subsequent round. Additionally, each base classifier is assigned a weight associated with the overall accuracy. The output of the testing sample is the weighted vote of each of the base classifiers. In this study, a modified version of AdaBoost is used. First, random sample selection is performed only on the negative samples (non-binding residues). All positive samples are used in each round because the number of negative samples is several orders of magnitude larger than the number of positive samples, especially for small ligands. Second, to prevent over-fitting and make full use of the negative samples, the weight of the misclassified negative samples increases on a small scale. The overall modified AdaBoost is shown in algorithm 1.
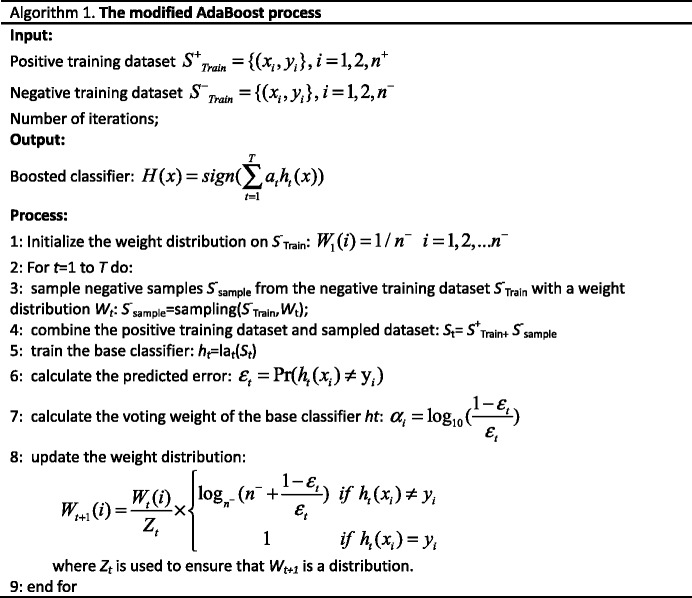


### Combination of the template-free and template-based methods

The proposed TargetCom method combines the template-free method (TargetSeq) and the template-based method (COFACTOR, TM-SITE, S-SITE and COACH) to get an improved performance (Fig. 1b). The process is similar to the proposed sequence-based method. A sliding window centred at the target residue is used to collect the output of each individual method. The target residue is then converted into a feature vector by concatenating the output of all residues in the window. The modified AdaBoost algorithm is then used to get the ensemble classifier which is then used to get the probability output for a testing residue. The overall flowchart is depicted in Fig. 1b.

Template-based methods use proteins with known ligand binding sites to infer the binding residues of the target sequence. The basic assumption behind these methods is that homologous proteins often have similar functions. Template-based methods have attracted a great deal of attention and shown a powerful performance in CASP [11]. However, the similarities between the target sequence and the template can affect the accuracy of the template-based methods. If no homologous templates are available for the “hard” target protein, the template-based methods will fail. In contrast, the template-free methods are robust because they use only sequence information, although the performance of the template-free methods is worse than the performance of the template-based methods when homologous templates can be identified. Based on this observation, we presented a combined method named TargetCom that combined the sequence-based and template-free method TargetSeq with four template-based methods (COFACTOR [16], TM-SITE, S-SITE and COACH [40]).

COFACTOR is a structure-based method that first uses a global structural alignment to identify possible templates with the same fold and then adopts the local 3D motif alignment to obtain the binding residues. TM-SITE uses a similar architecture but adds an additional clustering step to derive the binding sites. S-SITE uses a binding site-specific sequence profile-profile comparison to detect the templates and ligand binding sites. COACH is a consensus method that combines the output of the above three methods and two other methods and achieves a magnificent Continuous Automated Model EvaluatiOn (CAMEO) performance. To provide an unbiased comparison with the sequence-based method, all of the structure-based methods use a predicted model and are run in “benchmark” mode, in which all homologous templates with sequence identities larger than 30% are removed.

The probability output of the TargetSeq method is collected as one of the features of the TargetCom method. The C-score and cluster density of the other four methods are selected as input features. The C-score is the confidence score of the prediction and is calculated based on the similarity between the query target and the templates. The cluster density is the percentage of templates in specific binding sites. Because the proposed combination method is ligand-specific, the binding site predictions for specific ligands need to be extracted from the other four general-purpose methods. The possible ligands of the predicted binding site are collected by the identified templates. If one ligand matches the specific ligand, the binding site is selected as a candidate. This methodology is better than the method that only uses the most possible ligand (data not shown).

These features are also inputted to support vector machine to obtain the model. Then, the trained model is used to classify new testing samples.

### Evaluation metrics

The following metrics are used to evaluate the proposed methods: accuracy, sensitivity, specificity and the Matthews correlation coefficient (MCC).

5 $$ Accuracy=\frac{TP+TN}{TP+FP+TN+FN} $$

6 $$ Sensitivity=\frac{TP}{TP+FN} $$

7 $$ Specificity=\frac{TN}{TN+FP} $$

8 $$ MCC=\frac{TP\times TN-FP\times FN}{\sqrt{\left(TP+FP\right)\left(TP+FN\right)\left(TN+FP\right)\left(TN+FN\right)}} $$

where TP is the number of binding sites correctly predicted as binding residues, TN is the number of non-binding residues correctly predicted as non-binding residues, FP is the number of non-binding residues wrongly predicted as binding residues, and FN is the number of binding residues wrongly predicted as non-binding residues.

## Results and discussion

### Sequence-based method results

The proposed method (TargetSeq) was evaluated using five-fold cross-validation and compared with the S-SITE method. Although S-SITE is a template-based method, it does not use three-dimensional structure information. Therefore, here the comparison is performed on two sequence-based methods (the template-free method and the template-based method).

As shown in Table 2, the optimal window length of each ligand is different, with the small ligands usually having short window lengths and vice versa. The size of the binding pocket is generally proportional to the volume of the binding ligand; thus, the local neighbour information used to predict the binding residues might also change with the size of the binding ligand. The proposed method (TargetSeq) can make predictions for most ligands with an accuracy varying from 96.62 to 99.02%, specificity from 95.26 to 99.81% and MCC from 0.19 to 0.66. The performance on the SO4 ligand appeared to be especially low. As shown in the Additional file 1, none of the methods obtained a good performance on this ligand, indicating that SO4 was a hard ligand to predict. Overall, the proposed method outperformed the S-SITE method on most of the ligands with the exceptions of ATP and HEME, possibly because the large window length on these ligands introduced extra noise.

**Table 2:** Performance of the proposed sequence-based methods on the 9 types of ligands over five-fold cross-validation and comparison with S-SITE

Ligand	*w* ^a^	Method	Accuracy (%)	Sensitivity (%)	Specificity (%)	MCC
CU	15	TargetSeq	99.02	51.40	99.69	0.59
		S-SITE	97.98	60.37	98.50	0.46
FE	9	TargetSeq	98.83	53.54	99.52	0.57
		S-SITE	96.93	59.55	97.49	0.38
FE2	9	TargetSeq	99.20	51.36	99.81	0.63
		S-SITE	98.28	42.14	99.00	0.37
ZN	11	TargetSeq	99.01	41.78	99.68	0.50
		S-SITE	97.71	56.43	98.20	0.38
SO4	13	TargetSeq	97.79	10.07	99.66	0.19
		S-SITE	96.98	14.4	98.73	0.15
PO4	7	TargetSeq	98.09	20.18	99.59	0.31
		S-SITE	97.29	27.86	98.63	0.27
ATP	19	TargetSeq	97.14	36.81	99.31	0.48
		S-SITE	96.73	48.09	98.48	0.49
FMN	17	TargetSeq	97.23	56.59	99.32	0.66
		S-SITE	96.39	66.56	97.92	0.62
HEME	17	TargetSeq	92.62	61.27	95.26	0.53
		S-SITE	93.63	58.24	96.61	0.55

^a^The optimal window length

### Combined method results

The proposed combination method (TargetCom) combines the output of the proposed template-free method and four other template-based methods. COACH is also a consensus method and outperforms other methods, as shown in reference [40]. Therefore, we only list the comparison results of TargetCom and COACH in Table 3. The detailed results of all methods are provided in the Additional file 1.

**Table 3:** Performance of the proposed combined methods on the 9 types of ligands over five-fold cross-validation and comparison with COACH

Ligand	Method	Accuracy (%)	Sensitivity (%)	Specificity (%)	MCC
CU	TargetCom	99.21	57.94	99.78	0.67
	COACH	98.86	61.12	99.39	0.59
FE	TargetCom	98.73	59.73	99.32	0.58
	COACH	97.95	66.82	98.42	0.50
FE2	TargetCom	99.27	67.73	99.68	0.70
	COACH	99.20	62.41	99.67	0.66
ZN	TargetCom	98.99	56.18	99.50	0.56
	COACH	98.65	57.38	99.14	0.50
SO4	TargetCom	97.72	15.11	99.48	0.23
	COACH	97.21	19.15	98.87	0.21
PO4	TargetCom	97.99	32.03	99.26	0.37
	COACH	97.52	35.33	98.72	0.34
ATP	TargetCom	97.17	59.26	98.54	0.58
	COACH	96.99	56.27	98.46	0.55
FMN	TargetCom	97.66	79.61	98.58	0.76
	COACH	96.75	70.36	98.11	0.66
HEME	TargetCom	94.96	69.92	97.07	0.66
	COACH	94.48	61.60	97.25	0.60

**Fig. 2:**
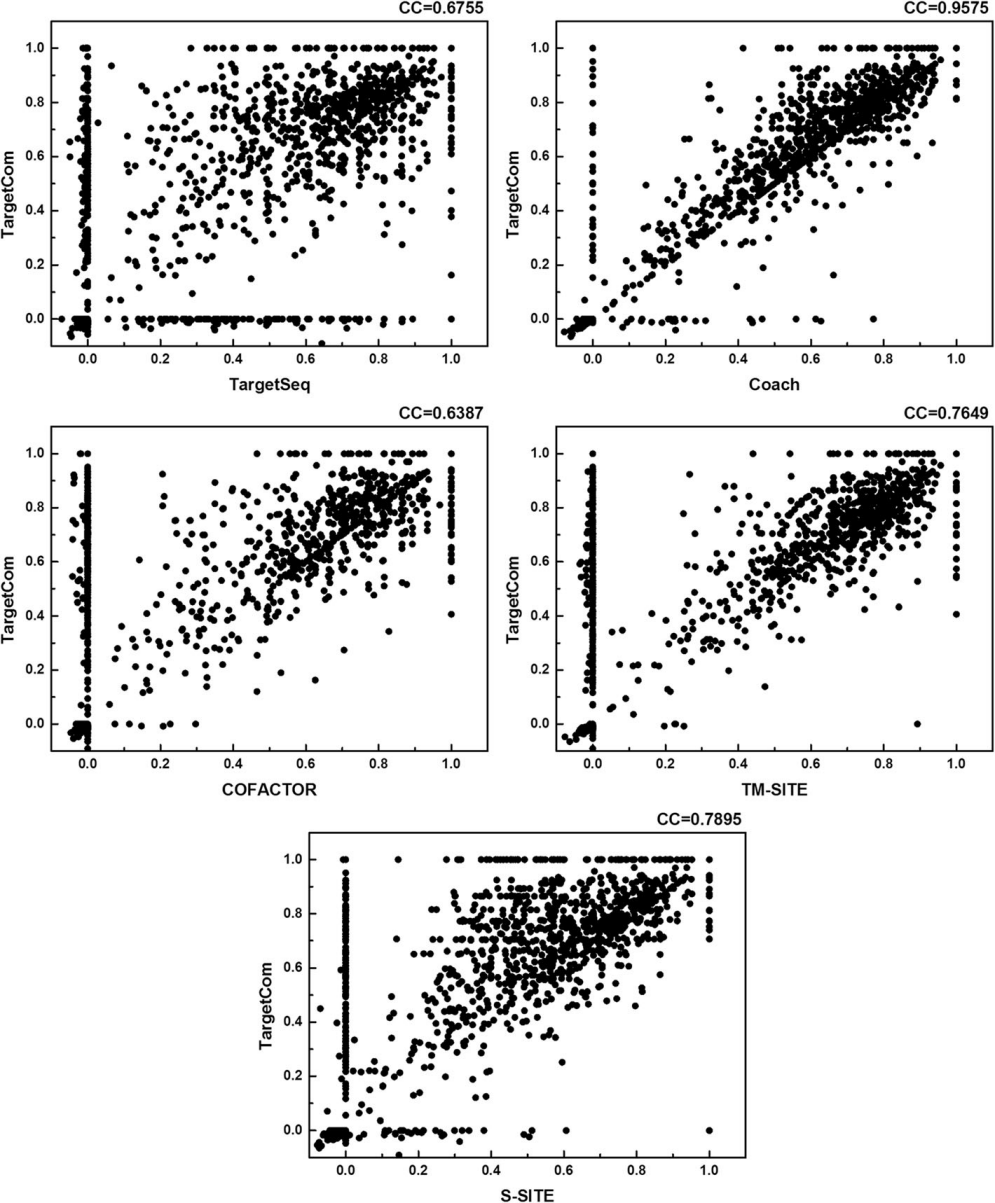
Head-to-head comparisons between TargetCom and the individual component methods on the proteins of all ligands. CC is the Pearson’s correlation coefficient between the MCCs of the two compared methods

The proposed TargetCom outperformed COACH on all ligands with an average MCC value increase of 0.0533, which was on average 10% higher than the COACH MCC value. The improvement made by TargetCom is mainly a result of the complement properties of the individual component predictor, as demonstrated by a previous study [40]. The template-free method is a complement of the template-based method that will be discussed in the subsequent section. The head-to-head comparison of TargetCom with the other individual methods is shown in Fig. 2. The Pearson correlation coefficient is also provided in the figure. The maximum correlation is observed between TargetCom and COACH, indicating that COACH makes the greatest contribution to TargetCom, followed by S-Site, TM-Site, TargetSeq and COFACTOR. The *P*-values of Student’s *t*-test between any two methods on the proteins of all ligands are calculated and shown in Table 4. The *P*-values between TargetCom and the other methods are all very small, demonstrating that the improvement from consensus is significant.

**Table 4:** The *p*-values in Student’s *t*-test for the differences in the MCC scores between each pair of predictors on the proteins of all ligands

Method	TargetCom	TargetSeq	COACH	COFACTOR	TM-SITE
TargetSeq	8.17562E-32				
COACH	2.09639E-44	1.17438E-10			
COFACTOR	4.366E-117	4.27612E-34	5.84742E-79		
TM-SITE	2.62453E-79	2.29774E-06	6.07923E-42	2.70076E-15	
S-SITE	7.1097E-112	2.47659E-10	5.31899E-68	7.66908E-11	0.0924092

### Data difference between BioLip and LPC

The first step towards the automatic prediction of ligand binding sites is defining the binding residues between the protein and ligand. Another important issue is that biologically irrelevant ligands need to be filtered before the ligand binding residues are identified. BioLip [44] is a newly developed, semi-manually curated database for biologically relevant ligand-protein interactions. The definition of a binding site is the same as the official CASP definition: a binding site is defined by all protein residues in the target structure having at least one (non-hydrogen) atom within a certain distance (*d*_*ij*_) of biologically relevant ligand atoms:

9 $$ {d}_{ij}<={r}_i+{r}_j+c $$

where *d*_*ij*_ is the distance between a residue atom *i* and a ligand atom *j, r*_*i*_ and *r*_*j*_ are the van der Waals radii of the involved atoms, and *c* is a tolerance distance of 0.5 Å.

Many previous studies used the Ligand Protein Contact (LPC) software [52] to define the binding residues; this software is based upon the surface complementarity analysis [53].

In this study, the difference between the binding sites defined by LPC and BioLip was investigated. The ATP168 dataset [32] is a representative dataset defined by LPC that is collected by Chauhan et al. for ATP binding site prediction. The same proteins are extracted from the BioLip database, and the corresponding binding sites of the ATP ligand are gathered. The binding sites of these proteins are compared using LPC and BioLip. We observed that the difference was significant. A total of 1968 common binding residues were defined by both methods. A total of 1117 binding residues were defined solely by LPC, and 208 binding residues were defined solely by BioLip. The number of binding residues defined by LPC was more than 40% higher than the number defined by BioLip. To quantitatively assess the influence of the binding site definition on the performance of the predictor, a base-line method (SVM-PSSM) that uses only PSSM as input for support vector machine is implemented and tested on the ATP168 dataset with different ligand binding site definitions. As shown in Table 5, the SVM-PSSM method with binding sites defined by the LPC database achieves performed significantly better than the method using the BioLip database. Because the method and the data are the same, this huge difference is definitely caused by the different binding site definitions. Because the LPC database defines more binding sites, the performance of the predictor trained on the LPC-derived dataset will be over-estimated.

**Table 5:** Performance comparison of SVM-PSSM on the ATP168 dataset with different definitions of ligand binding sites

Definition^a^	Accuracy (%)	Sensitivity (%)	Specificity (%)	MCC
LPC	96.00	33.40	99.28	0.47
BioLip	95.14	22.34	98.10	0.24

^a^Binding sites were defined using the LPC and BioLip databases, respectively

### The sequence-based method is a complement of the structure-based method

The structure-based method uses three-dimensional structures to identify binding sites, which can usually obtain better predictions than other methods. However, the structure-based methods will fail when no structures or homologous templates are available. In this case, sequence-based methods may be helpful, which will be quantitatively assessed here. The “hard” target proteins, which do not have any homologous templates, are identified by the multi-threading programme LOMETS [26]. For each threading program, the target-template alignment is measured by the Z-score, which is defined as the difference between the raw alignment score and the mean in the unit of derivation. A target protein is classified as “hard” if none of the threading programmes identifies a template with a Z-score larger than the specific threshold. The performance of all methods used in this study on the “hard” target proteins is listed in Table 6. As expected, none of the methods generated satisfactory predictions using these hard target proteins. Among the non-combined methods, the sequence-based methods (S-SITE and TargetSeq) significantly outperformed the structure-based methods (COFACTOR and TM-SITE). In most cases, the structure-based methods could not identify any binding sites. S-SITE usually performs better than the other methods on small ligands (CU, FE and ZN). TargetSeq performs better than the other methods on the ATP and PO4 ligands. These results demonstrate that the sequence-based methods are effective complements of the structure-based methods when no homologous templates are available.

**Table 6:** Performance of all methods on the “hard” target proteins over each type of ligand

Ligand	N^a^	Method	Accuracy (%)	Sensitivity (%)	Specificity (%)	MCC (%)^b^
CU	3	TargetCom	98.76	16.67	1	23.42
		COACH	98.76	16.67	1	23.42
		S-SITE	98.76	33.33	99.56	**27.04**
		TargetSeq	98.33	0	1	0
		COFACTOR	98.33	0	1	0
		TM-SITE	98.33	0	1	0
FE	3	TargetCom	98.48	16.67	1	23.39
		COACH	98.48	16.67	1	23.39
		S-SITE	98.73	25.00	1	**28.76**
		TargetSeq	98.26	12.5	99.9	20.03
		COFACTOR	97.98	0	1	0
		TM-SITE	97.98	0	1	0
ZN	30	TargetCom	98.38	40.63	99.42	37.49
		COACH	97.65	40.91	98.66	31.86
		S-SITE	97.43	43.32	98.37	**33.51**
		TargetSeq	97.97	7.02	99.68	11.43
		COFACTOR	98.14	0	1	0
		TM-SITE	97.99	0	99.85	−0.15
SO4	5	TargetCom	97.11	6.67	99.61	7.61
		COACH	97.02	6.67	99.51	7.08
		S-SITE	97.11	0	1	0
		TargetSeq	97.11	0	1	0
		COFACTOR	97.39	6.67	99.9	1
		TM-SITE	97.02	6.67	99.51	**7.08**
PO4	8	TargetCom	97.68	4.17	99.59	4.52
		COACH	97.54	05	99.43	2.9
		S-SITE	97.81	0	99.76	−0.36
		TargetSeq	98.08	13.39	99.66	**12.98**
		COFACTOR	97.91	0	99.86	−0.17
		TM-SITE	98.05	0	1	0
ATP	4	TargetCom	93.92	5	97.14	1.38
		COACH	93.92	5	97.14	1.38
		S-SITE	96.42	0	1	0
		TargetSeq	97.32	27.08	99.85	**33.79**
		COFACTOR	96.42	0	1	0
		TM-SITE	93.69	5	96.9	1.19
HEME	9	TargetCom	92.74	16.7	99.21	25.02
		COACH	91.8	03.98	99.3	07.3
		S-SITE	92.43	14.9	99.2	**17.25**
		TargetSeq	89.54	15.47	95.68	11.59
		COFACTOR	92.12	0	1	0
		TM-SITE	92.12	0	1	0

^a^The number of “hard” target proteins in each type of ligand

^b^The numbers shown in bold are the best values of the non-combination based method

### Ligand-specific method helps improve the prediction performance

The ligand-specific method trains models for each type of ligand, whereas the general purpose methods only use one model for all types of ligands. We will experimentally demonstrate the different performances of these strategies.

The datasets for all 9 ligands are merged into one single dataset. The positive samples are the binding residues regardless of the type of ligands to which they bind. The negative samples are the non-binding residues. The general purpose method is evaluated using this dataset via five-fold cross-validation. To give an unbiased comparison, the proposed TargetSeq method is re-performed on the merged dataset by cross-validation. During the evaluation phase, the performance is calculated for each type of ligand and compared with the ligand-specific mode of TargetSeq. As shown in Table 7, the ligand-specific mode of TargetSeq consistently outperforms the general purpose mode of TargetSeq in terms of accuracy, specificity and MCC. The performance of the general purpose mode decreases dramatically on small ligands. The sensitivities of the general purpose mode are higher than those of the specific mode, indicating that the general purpose mode of TargetSeq predicts too many binding residues. As expected, the average precision is only 13.39%. The precision is the percentage of correct predictions over all predictions.

**Table 7:** Performance comparison of the general purpose and ligand-specific models of the TargetSeq method on the dataset of the 9 ligands by five-fold cross-validation

Ligand Type	Model Type	Accuracy (%)	Sensitivity (%)	Specificity (%)	MCC
CU	General	86.98	79.62	87.09	0.22
	Specific	99.02	51.40	99.69	0.59
FE	General	90.14	85.02	90.22	0.29
	Specific	98.83	53.54	99.52	0.57
FE2	General	90.67	90.89	90.67	0.30
	Specific	99.20	51.36	99.81	0.63
ZN	General	88.50	74.29	88.66	0.27
	Specific	99.01	41.78	99.68	0.50
SO4	General	85.85	55.29	86.50	0.17
	Specific	97.79	10.07	99.66	0.19
PO4	General	86.38	71.73	86.66	0.23
	Specific	97.29	27.86	98.63	0.27
ATP	General	87.46	71.88	88.02	0.32
	Specific	96.73	48.09	98.48	0.49
FMN	General	88.24	76.68	88.83	0.40
	Specific	96.39	66.56	97.92	0.62
HEME	General	86.18	73.85	87.21	0.43
	Specific	93.63	58.24	96.61	0.55

### Comparison with other methods

There are many outstanding studies on ligand binding site prediction of proteins. The performance of the proposed methods is compared with that reported in other studies. ATP is one of the most extensively studied ligands for binding site prediction. The proposed TargetCom method achieves an overall accuracy of 97.17% and MCC value of 0.58 and the proposed TargetSeq method achieves an overall accuracy of 97.14% and MCC value of 0.48 on ATP ligand. The ATPsite method [31] reported an overall accuracy of 96.2% and MCC value of 0.43 which is lower than the proposed methods. The nSITEpred method [30] predicted the binding site for several nucleotides. They reported an overall accuracy of 96% and MCC value of 0.46 for ATP ligand, which is also lower than the proposed methods. The newly developed ATPBR method [54] reported an overall accuracy of 87.53% and MCC value of 0.55, where the accuracy is lower than the proposed methods, and the MCC value is larger than the TargetSeq method but lower than the TargetCom method.

Lu et al. [55] predict the binding sites of metal ions by using fragment transformation method. There are three metal ions (CU, FE2 and ZN) overlapped with the current study. They used accuracy, true positive rate and false positive rate as the evaluation metrics, so we use the accuracy as the compared metric. Lu et al. reported the accuracy of 94.9%, 94.9 and 94.8% for ligand CU, FE2 and ZN respectively, while the proposed TargetSeq method achieves the accuracy of 99.02%, 99.20 and 99.01% and the proposed TargetCom method gets the accuracy of 99.21%, 99.27 and 98.99% for ligand CU, FE2 and ZN respectively. It is clearly show that the proposed methods outperform the method of Lu et al.

The HemeBIND method [33] predict the binding sites of HEME ligand and reported an overall accuracy of 97.17% and MCC value of 0.58. The proposed TargetCom method achieves an overall accuracy of 94.96% and MCC value of 0.66 and the proposed TargetSeq method achieves an overall accuracy of 92.62% and MCC value of 0.53 on HEME ligand.

The above comparison shows that the proposed methods provide the state-of-the-art performance for binding site prediction of proteins.

## Conclusion

This study presented two effective ligand-specific methods for ligand binding site prediction. The sequence-based method uses only sequence information and adopts the improved AdaBoost method for binding site predictions. The combined method combines the template-free and template-based methods. Both methods are tested on the dataset extracted from the recently developed, semi-manually curated ligand binding site database (BioLip). The experimental results demonstrate the efficacy of the proposed methods. The sequence-based method is an effective complement to the structure-based method when no structures are available or no homologous templates can be identified. The ligand-specific methods can help improve the prediction performance. We also found that the binding site definition in BioLip was stricter than the definition in LPC.

Future directions are to use a feature selection or extraction algorithm to remove the possible noise in the high dimensional feature space. Another issue for ligand-specific binding site prediction is how to select the negative sample (non-binding residues) because proteins may have multiple ligands. The non-binding residues for one ligand may be binding residues for another ligand; thus, these residues have potential binding ability. The ligand-specific predictor needs to be intensively explored to develop an excellent method for ligand binding site prediction.

## Additional file

Additional file 1: Table S1. Performance of all methods used in the paper on 9 types of ligands. (DOCX 20 kb)
