# Supplementary material for: Protein ligand-specific binding residue predictions by an ensemble classifier
Source: BMC Bioinformatics. 2016 Nov 17;17:470. doi: 10.1186/s12859-016-1348-3 (PMC5114821; doi:10.1186/s12859-016-1348-3)
Supplement: Additional file 1: Table S1. — Performance of all methods used in the paper on 9 types of ligands. (DOCX 20 kb) [file 12859_2016_1348_MOESM1_ESM.docx]

**Additional file 1 for**

**“**Protein ligand-specific binding residue predictions by an ensemble classifier**”**

Table S1. Performance of all methods used in the paper on 9 types of ligands

| Ligand | Method | Accuracy | Sensitivity | Specificity | MCC |
| --- | --- | --- | --- | --- | --- |
| CU | TargetCom | **0.9921** | 0.5794 | **0.9978** | **0.6715** |
|  | TargetSeq | 0.9902 | 0.5140 | 0.9969 | 0.5928 |
|  | COACH | 0.9886 | **0.6112** | 0.9939 | 0.5901 |
|  | S-site | 0.9798 | 0.6037 | 0.985 | 0.4564 |
|  | COFACTOR | 0.9904 | 0.4019 | 0.9985 | 0.5607 |
|  | TM-site | 0.9904 | 0.471 | 0.9976 | 0.5830 |
| FE | TargetCom | 0.9873 | 0.5973 | 0.9932 | **0.5767** |
|  | TargetSeq | 0.9883 | 0.5354 | **0.9952** | 0.5732 |
|  | COACH | 0.9795 | **0.6682** | 0.9842 | 0.5009 |
|  | S-site | 0.9693 | 0.5955 | 0.9749 | 0.3835 |
|  | COFACTOR | **0.9854** | 0.3668 | 0.9947 | 0.4265 |
|  | TM-site | 0.9841 | 0.5937 | 0.9900 | 0.5214 |
| FE2 | TargetCom | **0.9927** | **0.6773** | 0.9968 | **0.7004** |
|  | TargetSeq | 0.9920 | 0.5136 | 0.9981 | 0.6299 |
|  | COACH | 0.9920 | 0.6241 | 0.9967 | 0.6607 |
|  | S-site | 0.9828 | 0.4214 | 0.9900 | 0.376 |
|  | COFACTOR | 0.9890 | 0.1822 | **0.9994** | 0.3749 |
|  | TM-site | 0.9919 | 0.5626 | 0.9974 | 0.6402 |
| ZN | TargetCom | 0.9899 | 0.5618 | 0.9950 | **0.5599** |
|  | TargetSeq | 0.9901 | 0.4178 | **0.9968** | 0.4994 |
|  | COACH | 0.9865 | **0.5738** | 0.9914 | 0.4952 |
|  | S-site | 0.9771 | 0.5643 | 0.9820 | 0.3794 |
|  | COFACTOR | **0.9912** | 0.3549 | 0.9986 | 0.5133 |
|  | TM-site | 0.9883 | 0.3641 | 0.9956 | 0.4190 |
| SO4 | TargetCom | 0.9772 | 0.1511 | 0.9948 | **0.2307** |
|  | TargetSeq | **0.9779** | 0.1007 | **0.9966** | 0.1879 |
|  | COACH | 0.9721 | **0.1915** | 0.9887 | 0.2114 |
|  | S-site | 0.9698 | 0.1440 | 0.9873 | 0.1525 |
|  | COFACTOR | 0.9762 | 0.1054 | 0.9948 | 0.1680 |
|  | TM-site | 0.9773 | 0.1167 | 0.9956 | 0.1963 |
| PO4 | TargetCom | 0.9799 | **0.3203** | 0.9926 | **0.3731** |
|  | TargetSeq | **0.9809** | 0.2018 | **0.9959** | 0.3061 |
|  | COACH | 0.9752 | 0.3533 | 0.9872 | 0.3381 |
|  | S-site | 0.9729 | 0.2786 | 0.9863 | 0.2667 |
|  | COFACTOR | 0.9786 | 0.2145 | 0.9933 | 0.2764 |
|  | TM-site | 0.9798 | 0.2408 | 0.9941 | 0.3163 |
| ATP | TargetCom | **0.9717** | **0.5926** | 0.9854 | **0.5786** |
|  | TargetSeq | 0.9714 | 0.3681 | 0.9931 | 0.4788 |
|  | COACH | 0.9699 | 0.5627 | 0.9846 | 0.5494 |
|  | S-site | 0.9673 | 0.4809 | 0.9848 | 0.4894 |
|  | COFACTOR | 0.9666 | 0.1517 | **0.9959** | 0.2828 |
|  | TM-site | 0.9703 | 0.4577 | 0.9888 | 0.5069 |
| FMN | TargetCom | **0.9766** | **0.7961** | 0.9858 | **0.7568** |
|  | TargetSeq | 0.9723 | 0.5659 | **0.9932** | 0.6642 |
|  | COACH | 0.9675 | 0.7036 | 0.9811 | 0.6625 |
|  | S-site | 0.9639 | 0.6656 | 0.9792 | 0.6240 |
|  | COFACTOR | 0.9691 | 0.5561 | 0.9903 | 0.6293 |
|  | TM-site | 0.9703 | 0.6385 | 0.9873 | 0.663 |
| HEME | TargetCom | **0.9496** | **0.6992** | 0.9707 | **0.6560** |
|  | TargetSeq | 0.9262 | 0.6127 | 0.9526 | 0.5254 |
|  | COACH | 0.9448 | 0.6160 | 0.9725 | 0.6046 |
|  | S-site | 0.9363 | 0.5824 | 0.9661 | 0.5522 |
|  | COFACTOR | 0.9446 | 0.5138 | **0.9808** | 0.5682 |
|  | TM-site | 0.945 | 0.5398 | 0.9791 | 0.5794 |
